# Sorting Overlapping Spike Waveforms from Electrode and Tetrode Recordings

**DOI:** 10.3389/fninf.2017.00053

**Published:** 2017-08-17

**Authors:** Yasamin Mokri, Rodrigo F. Salazar, Baldwin Goodell, Jonathan Baker, Charles M. Gray, Shih-Cheng Yen

**Affiliations:** ^1^Department of Electrical and Computer Engineering, National University of Singapore Singapore, Singapore; ^2^Department of Cell Biology and Neuroscience, Montana State University, Bozeman MT, United States

**Keywords:** spike sorting, overlapping waveforms, tetrode, visual cortex, electrophysiology

## Abstract

One of the outstanding problems in the sorting of neuronal spike trains is the resolution of overlapping spikes. Resolving these spikes can significantly improve a range of analyses, such as response variability, correlation, and latency. In this paper, we describe a partially automated method that is capable of resolving overlapping spikes. After constructing template waveforms for well-isolated and distinct single units, we generated pair-wise combinations of those templates at all possible time shifts from each other. Subsequently, overlapping waveforms were identified by cluster analysis, and then assigned to their respective single-unit combinations. We examined the performance of this method using simulated data from an earlier study, and found that we were able to resolve an average of 83% of the overlapping waveforms across various signal-to-noise ratios, an improvement of approximately 32% over the results reported in the earlier study. When applied to additional simulated data sets generated from single-electrode and tetrode recordings, we were able to resolve 91% of the overlapping waveforms with a false positive rate of 0.19% for single-electrode data, and 95% of the overlapping waveforms with a false positive rate of 0.27% for tetrode data. We also applied our method to electrode and tetrode data recorded from the primary visual cortex, and the results obtained for these datasets suggest that our method provides an efficient means of sorting overlapping waveforms. This method can easily be added as an extra step to commonly used spike sorting methods, such as KlustaKwik and MClust software packages, and can be applied to datasets that have already been sorted using these methods.

## Introduction

When recording extra-cellularly from multiple neurons, it is common to have action potential waveforms from one neuron altered by the action potentials of other neurons. This often results in waveform shapes and amplitudes that are significantly different from single-unit waveforms, thereby posing problems for spike sorting algorithms, especially when those algorithms are automated. This problem is exacerbated when using devices like electrode arrays (Normann et al., 1989), silicon electrodes (Anderson et al., 1989), and tetrodes (Wilson and McNaughton, 1993; Gray et al., 1995), which enable recording from large numbers of neurons simultaneously. Resolving these waveforms correctly into their constituent single units can be critically important for a range of analyses like response variability (Berry et al., 1997; de Ruyter van Steveninck et al., 1997), correlation (Maldonado et al., 2008; Cohen and Maunsell, 2009; Ecker et al., 2010), latencies (Keat et al., 2001; Gollisch and Meister, 2008), and information rates (Reich et al., 2000, 2001). Many attempts have been made to address this problem, but the resolution of overlapping waveforms is still not routinely performed in spike sorting. This could be due to limiting constraints of some of the available methods, some of which are described in the following paragraphs, or because of the high complexity of some other methods, which makes incorporating them into current spike sorting workflows difficult.

The most common approaches used to resolve overlapping waveforms in extracellular recordings create combinations of previously identified single-unit waveforms, usually referred to as templates, and then measure the similarity between the overlapping waveforms and the template combinations. These methods usually use the amplitude values of the waveforms to find the best match, although other features such as Fourier coefficients of the waveforms have also been used (Rinberg et al., 2003; Wang and Liang, 2005). Different techniques, such as different machine learning techniques, were used to compare the overlaps with the templates, such as support vector machines (SVM) (Ding and Yuan, 2008) and RELAX (Li and Stoica, 1996; Wang et al., 2009), a decoupled parameter estimation algorithm, that was used by Wang and Liang (2005). Also, Zhang et al. (2004) subtracted the templates from the overlaps, and used the similarity of the residue with a Gaussian distribution to find the best match, as they assumed the noise distribution was Gaussian. A similar method was used by Vargas-Irwin and Donoghue (2007). In all these techniques, a certain threshold needs to be determined to find the best matching template. Template-matching methods usually tend to be slow as well, because comparisons of all potential overlapping waveforms with all template combinations are needed. Recently, Adamos et al. (2010), addressing most of these issues, used a neural network to match overlapping waveforms with the templates generated by superimposing single-unit templates. However, it seems that the capability of this neural network in rejecting waveforms that do not belong to any of the identified single units, or combinations of those single units, remains to be investigated.

A very similar approach to Zhang et al. (2004) and Vargas-Irwin and Donoghue (2007) was taken by Prentice et al. (2011) and Pillow et al. (2013), in which they also subtracted the best matching spikes from the recorded signal until the residue was indistinguishable from noise. The difference was that these methods used greedy algorithms to find the match instead of using a brute-force search. This meant they will scale better. However, both these methods needed to make assumptions about factors (such as the distribution of noise or spike trains) that are simplified. For example, Prentice et al. (2011) assumed a Poison distribution for the firing of the cells, which is reasonable, but may not be applicable in a number of cases (Berry et al., 1997; Berry and Meister, 1998). As mentioned by both these studies, adding more constraints to make these assumptions more similar to real recordings may make these methods computationally expensive.

Other methods that do not involve template matching concentrated on extracting more robust features other than amplitude to cluster overlapping waveforms with single-unit waveforms. Among these methods, Hulata et al. (2002) and Quiroga et al. (2004) represented the waveforms using wavelet coefficients, and used k-means clustering and superparamagnetic clustering, respectively. However, they appear to have trouble resolving overlapping waveforms resulting from the near simultaneous firing of multiple neurons.

Another group of methods aimed to decompose the overlaps into their components. For example, in Oweiss and Anderson (2007), the overlapping waveforms were decomposed using the discrete wavelet packet transform, Takahashi et al. (2003) applied the independent components analysis (ICA) technique, and Franke et al. (2010, 2015) used a set of linear filters to decompose the overlapping waveforms. For these methods, like other methods discussed earlier, one needs to set a threshold to measure the similarity. Franke et al. (2010, 2015) used an analytical method to find the threshold, but it seems that to use this method, it is necessary to assume a Gaussian distribution for noise, as well as the distribution of single-unit spikes. Moreover, the other drawback of the method described in Takahashi et al. (2003) and Franke et al. (2010, 2015) is that two single units with different amplitudes but similar amplitude patterns cannot be distinguished from each other, although this could be advantageous in dealing with bursting cells. Ekanadham et al. (2014), who used Continuous Basis Pursuit in order to estimate the most probable spike patterns given the observed recording, also suffered from the same drawback, although their method has the advantage that it can be scaled for sorting multi-electrode array recordings.

More recently, methods developed for use with high density multi-electrode arrays, with much smaller spacing between electrodes, have taken advantage of the fact that the activity of one neuron appears on several different electrodes, and the spatial information provided by these arrays can be used to resolve overlapping waveforms (Marre et al., 2012; Pachitariu et al., 2016; Rossant et al., 2016; Yger et al., 2017; see review by Lefebvre et al., 2017). However, a lot of recordings in larger animals, and in human subjects, are still performed with electrode arrays with electrode spacing larger than 200 μm (e.g., Blackrock Utah Array, Microprobe Floating Microelectrode Arrays, etc.). These electrode separations greatly reduce the likelihood of multiple electrodes recording the activity of one neuron, and thus negates some of the advantages that these methods provide.

In order to eliminate some of these difficulties, we propose a partially automated method to resolve overlapping waveforms based on template matching. Our method uses KlustaKwik to perform the clustering, and the MClust software package to visualize and inspect the results of the spike sorting. As KlustaKwik is among the most popular automated clustering methods used for spike sorting (Wild et al., 2012), and MClust is a widely used package for spike sorting, applying our method to currently sorted datasets may be less problematic compared to some other methods. Our method has been designed to be added as an extra step to spike sorting routines currently in use for single electrode and tetrode recordings, and has the advantage that it can be parallelized in order to decrease the computational load. In our method, all steps, except the single-unit template selection, are automated and unsupervised. We show that despite the simplicity of our automated approach, it can successfully produce spike trains with fairly high accuracy in simulated data.

## Materials and Methods

The method is comprised of the following main steps: (1) Finding the single unit templates; (2) Generating the overlapping waveform templates; (3) Clustering the non-single-unit spikes and the overlapping waveform templates together (to find the best matching overlapping waveform template for each overlapping spike); and (4) Assigning the identified overlapping spikes to the single unit template clusters based on the best matching overlapping waveform templates. We describe these steps in detail in the following sections.

### Spike Extraction

An amplitude thresholding method (Quiroga et al., 2004; Yen et al., 2007) was applied to extract the spikes. In summary, the local minima of the high-pass filtered data were identified if they exceeded a certain threshold, and 1 ms of data around each local minimum were isolated as a spike. This was 10 points before and 20 points after each minimum for the example datasets used in this paper, as the sampling rate for these datasets was 30 kHz. As a result, all spikes were aligned at the local minimum (point 11 for our examples), and this point will be referred to as the “trigger point” of a spike in the rest of the paper.

**FIGURE 1:**
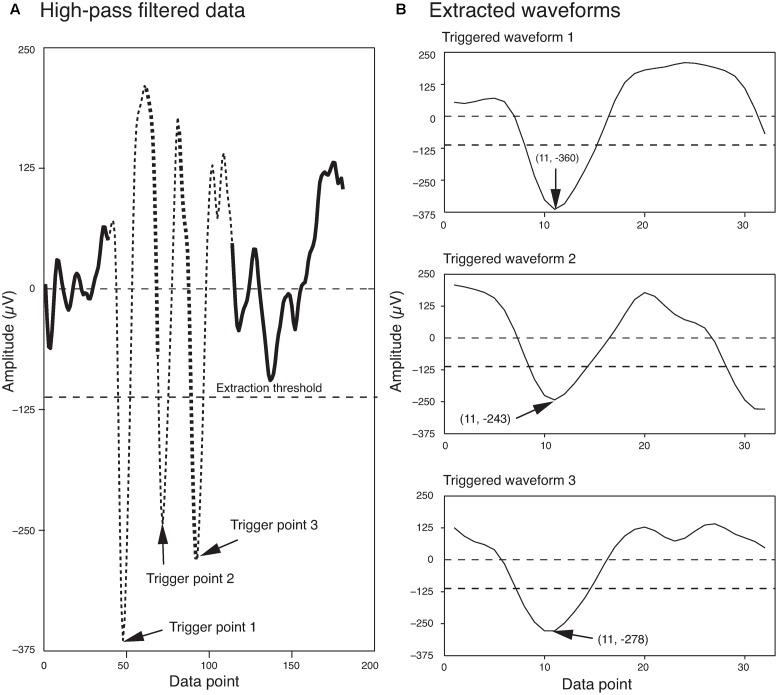
Waveform Extraction. **(A)** A short segment of the high-pass filtered data is shown here. Three local minima that exceeded the extraction threshold (dashed line) are highlighted by arrows. The extracted waveforms are indicated by thin broken lines. Regions of overlap between the extracted waveforms are indicated by the thick broken lines. **(B)** The three extracted waveforms corresponding to the three trigger points shown in **(A)**. The trigger points appear as data point 11 in each of the extracted waveforms. The first and second numbers in the parentheses indicate the data point number and the voltage, respectively.

The extraction threshold was set as a multiple of the standard deviation of the background noise. Although, four times the standard deviation of the background noise is commonly used in the field (Quiroga et al., 2004), sometimes this can be changed for a dataset based on the signal to noise ratio of the recorded signal. Potential errors associated with this spike extraction method are discussed in detail in Quiroga et al. (2004). **Figure 1** shows three spikes extracted from a segment of a single electrode recording, using this method.

### Clustering

For both identifying the single unit templates and overlapping waveforms, we used KlustaKwik (Harris et al., 2000), a clustering method that fits the data with a mixture-of-Gaussian model optimized using the classification expectation maximization (CEM) algorithm (Celeux and Govaert, 1992), a modified version of the expectation maximization (EM) algorithm. KlustaKwik is commonly used for spike sorting and can be easily used for clustering high dimensional data collected from multichannel recording electrodes (Kadir et al., 2014). KlustaKwik has a number of parameters, such as the minimum and maximum number of clusters, and a cost function that can be adjusted in order to improve the performance of the clustering for a particular dataset. As the focus of this paper is not on the advantages of using a particular clustering method, we will not describe the effect of adjusting these parameters here, but refer to Wild et al. (2012) for more details on the effect of varying these parameters, and also for a comparison between KlustaKwik and other state of the art clustering techniques commonly used for spike sorting. For the examples used in this paper, we used the default values for nearly all the parameters in KlustaKwik, except the minimum number of clusters (5 for electrode data and 30 for tetrode data), the maximum number of clusters (30 for electrode data, and 100 for tetrode data), and the cost function (Bayesian Information Cost for electrode data, and Akaike Information Cost for tetrode data). These parameters worked well in our experience, but were not chosen based on any objective measures.

In order to compute waveform features for clustering, we used MClust^1^, another popular software package. Techniques similar to what was proposed by Bestel et al. (2012) can be applied, to find the best feature set for a particular dataset. However, in practice in our lab, we selected the features by assessing the resulting clusters visually. It should be mentioned that KlustaKwik and the features that were used here can be replaced easily by any other clustering method or feature set, as our method is not dependent on a specific clustering method or waveform feature.

### Constructing the Single Unit Templates

As the first step in sorting the extracted spikes, we needed to identify the single units that contributed to forming the overlapping waveforms. Our method is based on template matching, and single units are used to generate templates that were subsequently matched with overlapping waveforms. In order to find the single units, we clustered all the spike waveforms using the method described earlier. The resulting clusters were subsequently inspected, and the largest and densest clusters with large isolation distances, a measure of the separation of a cluster from other points (Schmitzer-Torbert et al., 2005), were chosen as potential templates. However, sometimes clusters with large isolation distances contained only waveforms with very small amplitudes. These clusters were removed from consideration as template clusters, because they mostly looked like multi-unit activity. The inter-spike-interval histogram of each cluster was also inspected to reject clusters with large numbers of refractory violations (i.e., intervals smaller than 1 ms), as that implied that those clusters contained a mixture of waveforms from different cells. The candidates were visually inspected to ensure the clusters were made up of waveforms of different shapes. If a number of clusters exhibited similar waveforms, we merged these clusters together, as long as they did not add noise, or refractory period violations, to the template clusters.

**FIGURE 2:**
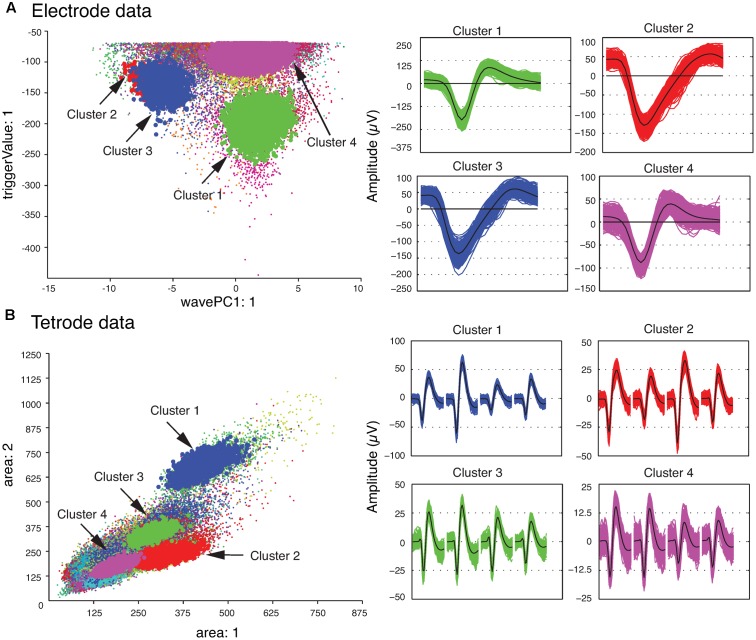
Clustered waveforms and candidate template clusters. **(A)** Electrode data. The features shown are the first principle component on the horizontal axis, and the voltage at the trigger point on the vertical axis. **(B)** Tetrode data. The features shown are the waveform area for the first channel on the horizontal axis, and waveform area for the second channel on the vertical axis. (Left) Initial clusters. Clusters with large isolation distances are highlighted in red, green, blue, and magenta. Isolation distances for the electrode data computed using the features shown were: Cluster 1 – 33.0; Cluster 2 – 0.85; Cluster 3 – 4.79; Cluster 4 – 7.23. For tetrode data, they were: Cluster 1 – 2.75; Cluster 2 – 0.84; Cluster 3 – 0.85; Cluster 4 – 1.13. (Right) Waveforms corresponding to highlighted clusters. For the tetrode data, the 32 data points from each of the four channels are plotted next to each other. The average waveform for each cluster is plotted in black.

We also checked the stability of the waveform features of the template clusters over time to ensure that only stable recordings were used in the subsequent analysis steps. This was easily accomplished by assessing the plots that showed each feature versus time using MClust. After the single unit template clusters were selected, the remaining clusters consisted of overlapping and multi-unit waveforms. We then computed the average of all the waveforms in each template cluster to construct the single-unit templates. These average waveforms will be referred to as single-unit templates for the rest of this paper. **Figure 2** shows four clusters with the largest isolation distances for each of the sample electrode and tetrode datasets. For the sample electrode data, using these four clusters, we generated two single unit template clusters. The cluster shown in magenta (Cluster 4) was comprised mainly of waveforms with small amplitudes. It also contained a sharp edge in the feature space that implied that some of the waveforms that belonged to this cluster were cut off by the extraction threshold. This was typical of multiunit activity, so it was not selected as a template cluster. On the other hand, the waveforms in Cluster 2 and Cluster 3 were very similar to each other, and as a result, we merged these two clusters into a larger single-unit template cluster. Cluster 1 was selected as the other template cluster. For the sample tetrode data, all four highlighted clusters shown in **Figure 2B** were selected as template clusters.

### Single Unit Template Superposition and Generation of Synthetic Waveforms

We then generated a set of templates for the overlapping spikes. These templates were subsequently matched with overlapping waveforms to identify the single units that contributed to each overlapping waveform.

**FIGURE 3:**
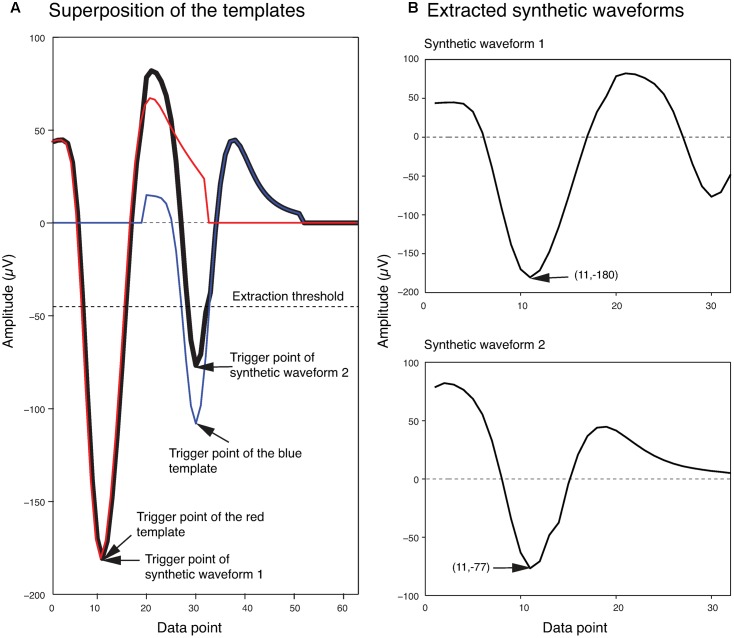
Extraction of synthetic waveforms. **(A)** Superposition (thick black line) of two templates (red and blue lines) with their trigger points separated by 19 data points. **(B)** Extracted synthetic waveforms. The data point number and amplitude of the trigger points are shown in parentheses.

In order to generate the templates for overlapping waveforms, first, for each pair of single unit templates, the two single unit templates were shifted relative to each other, and then were linearly added together point by point to create a superposition waveform corresponding to each shift. Since each single unit template was 32 data points long, the superposition waveforms consisted of different numbers of points, ranging from 32 points (when the two templates overlapped completely) to 63 points (when the two templates overlapped by just one point). In order to match these superposition waveforms to the 32-point overlapping waveforms, 32-point waveforms would have to be generated from these superposition waveforms. We did this by using the same amplitude thresholding procedure described earlier in the spike extraction section to extract 32-point waveforms from each superposition waveform. We referred to these waveforms extracted from the superposition waveforms simply as *synthetic waveforms*. This was to distinguish them from the real overlapping waveforms in the recordings, which we will simply refer to as overlapping waveforms. As an example, two synthetic waveforms that were extracted from the superposition of two templates at one phase shift are shown in **Figure 3**. In **Figure 3A**, the two templates (shown in blue and red) were superimposed with a phase shift of 19 data points to generate the superposition waveform. Two minima were then identified at data points 11 and 30 (labeled as trigger points of Synthetic Waveform 1 and Synthetic Waveform 2, respectively, in the figure). The 10 data points before each of these trigger points, and the 21 data points after, were extracted to form the synthetic waveforms shown in **Figure 3B**. It should be mentioned again here that these synthetic waveforms looked different from the single-unit templates as they were extracted from combinations of the original single-unit templates.

Similar to some other methods, such as Quiroga et al. (2004) and Herbst et al. (2008), we assumed it was unlikely that more than two neurons would fire simultaneously, and as a result the error associated with not sorting these overlaps would be small. Thus, in the current implementation of our method, we only considered pair-wise combinations of template waveforms. Although it may be possible to extend our method to sort overlaps generated as a result of synchronous firing of more than two cells, it is not straightforward because of the significant increase in the number of combinations, and the increase in likelihood that different combinations can create similar overlapping waveforms.

### Matching of Synthetic Waveforms with Overlapping Waveforms

**FIGURE 4:**
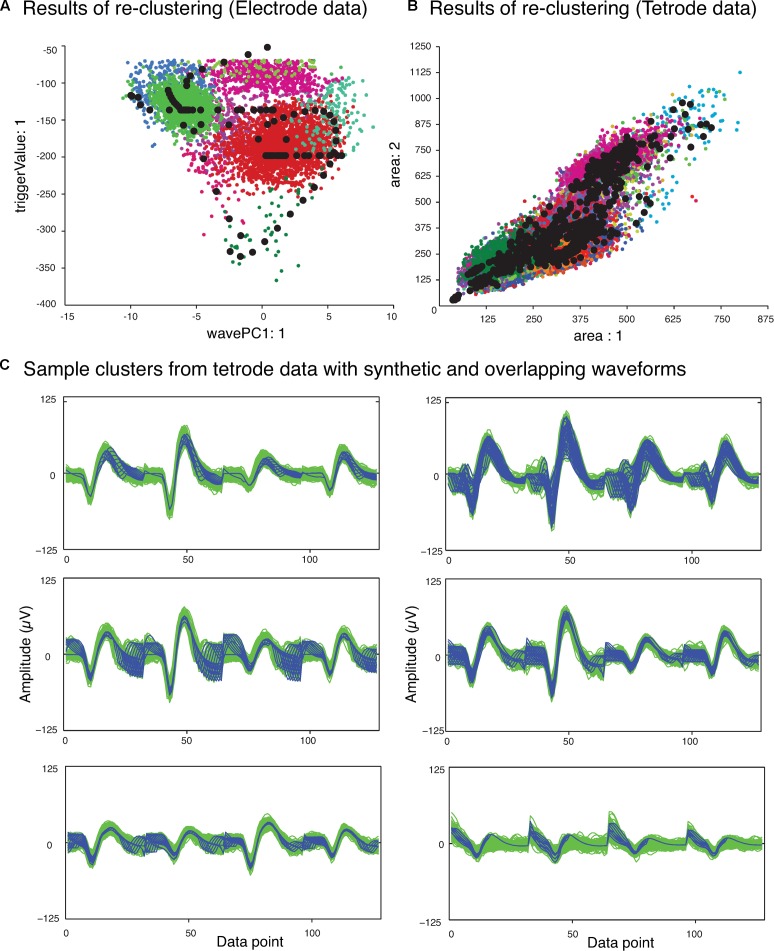
Results of second clustering of data shown in **Figure 2**. **(A)** Electrode data. **(B)** Tetrode data. Synthetic waveforms are highlighted as black circles. These waveforms were clustered with real overlapping waveforms. **(C)** Waveforms from six clusters that included both synthetic (blue) and overlapping waveforms (green) for the tetrode data. Two distinct minima can be seen on each channel corresponding to the single spikes that contributed to the overlap.

The next phase of the template matching procedure was the comparison of the overlapping waveforms with all the synthetic waveforms generated from combining the single unit templates. In order to perform this, we developed a method based on clustering. We grouped all the recorded spike waveforms that did not belong to the template clusters with the synthetic waveforms, and then re-clustered this new set of waveforms using KlustaKwik. We assumed that the overlapping waveforms and their best matching synthetic waveforms would be clustered together, and this method would be more accurate for template matching, as we would be able to use more than one feature to compare the overlapping waveforms with available templates, compared to solely using amplitude. Also this allowed us to avoid using iterative subtraction of the templates from the overlapping waveforms, which Lewicki (1998) pointed out might lead to matching errors due to the noise generated by the inaccuracies of the templates. The results of the second clustering for the sample electrode and tetrode datasets shown earlier (**Figure 2**) are shown in **Figures 4A,B**, respectively. Waveforms from six of the clusters that included both synthetic and overlapping waveforms for the tetrode data are also shown (**Figure 4C**).

Features we used for re-clustering of the sample electrode data were area, trigger value, Fast Fourier Transform (FFT), and the first principal component of the waveform. For the sample tetrode data, we used area, trigger value, FFT, the first principal component of the waveform, spike width, and peak value. It should be mentioned again that we empirically found that these features gave us better clustering results, but these features may vary from dataset to dataset.

**FIGURE 5:**
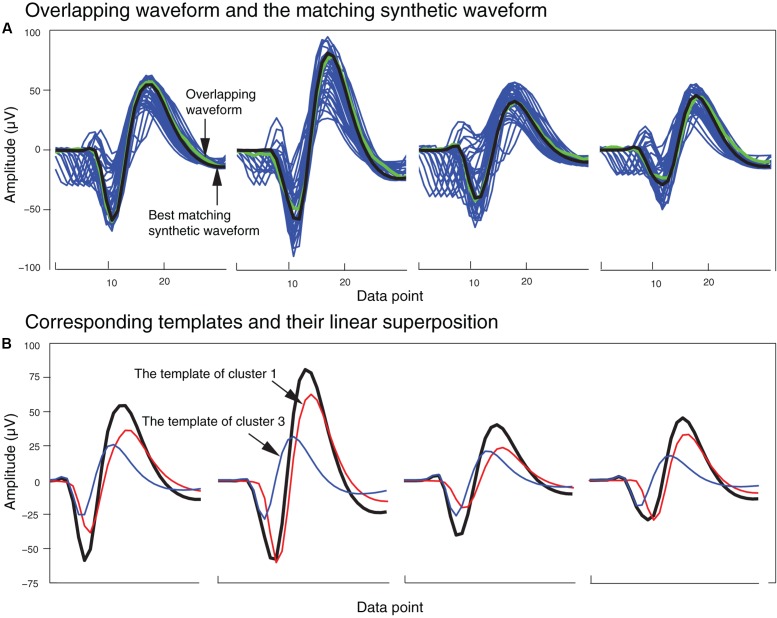
Matching of synthetic and overlapping waveforms for the tetrode data recording shown in **Figures 2**, **4**. **(A)** The synthetic waveforms (blue) clustered with one overlapping waveform (green) are shown with the best-matched synthetic waveform highlighted in black. **(B)** The template waveforms (blue and red) that generated the synthetic waveform (black) shown in **(A)**. The cluster numbers refer to the cluster numbers in **Figure 2B**.

In order to identify the best matching synthetic waveform for an overlapping waveform, we computed the Pearson correlation coefficient for all possible pairs of synthetic and overlapping waveforms inside each of the clusters that included both synthetic and overlapping waveforms. The synthetic waveform with the highest correlation coefficient was selected as the best match, and used in the next stage to determine how the overlapping waveform would be assigned to one or both of the single unit template clusters that formed that synthetic waveform. We also attempted using other similarity measures like sum-squared error, but found that the correlation coefficient returned the best overall results. In **Figure 5A**, an overlapping waveform (green), from the sample tetrode recording shown in **Figures 2**, **4**, is shown along with the synthetic waveform (black) with the highest correlation coefficient. Other synthetic waveforms in the same cluster are shown in blue for reference. It is important to note that if we had used only the correlation coefficient to find the best match when comparing an overlapping waveform with all possible synthetic waveforms, this could have incorrectly grouped waveforms with similar time courses but different amplitudes. However, since the correlation coefficients were computed only on waveforms that were clustered together using both amplitude and shape features, this significantly reduced the possibility of errors using the correlation coefficient.

### Assignment

**FIGURE 6:**
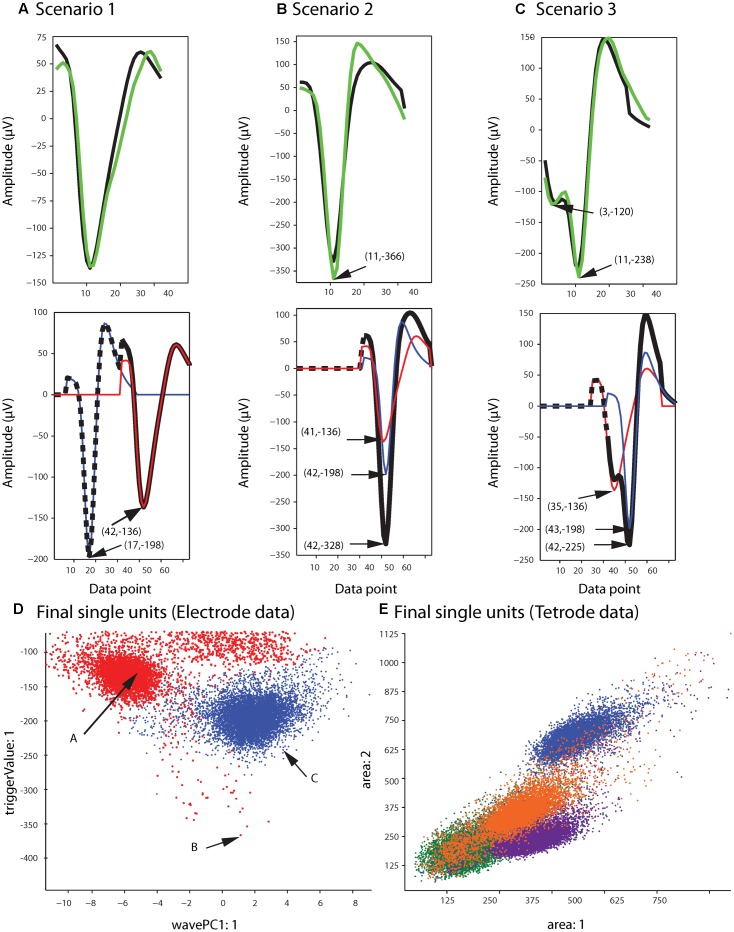
Waveform assignment. **(A–C)** The different scenarios under which overlapping waveforms were assigned are illustrated. (Top) Overlapping waveforms (green) are shown along with their best-matched synthetic waveforms (black). (Bottom) The best-matched synthetic waveform (black) was extracted from the superposition waveform (dotted and thick black line) that was the result of adding the two template waveforms (blue and red) together. The arrows in both the top and bottom plots highlight the trigger points of the overlapping, synthetic, and template waveforms. **(D,E)** Final single-unit clusters after assigning the overlapping waveforms in the electrode **(D)** and tetrode data **(E)**. The data shown is the same as in **Figures 2**, **4**. The arrows in **(D)** indicate the positions of the overlapping waveforms shown in **(A–C)** in the waveform feature space.

**Figure 5B** shows the single unit templates and the corresponding phase shift between these templates that generated the synthetic waveform with the highest correlation coefficient in **Figure 5A**. It can be seen that these single unit templates had small phase shifts with respect to each other, and as a result were highly overlapped so that the resulting synthetic waveform looked like one single spike and had only one minimum. This means that in place of the overlapping waveform that was matched with this synthetic waveform, one spike must be added to each single unit template as the two spikes happened almost simultaneously at this time point. This scenario for assigning overlapping waveforms to their corresponding single units is illustrated with two additional scenarios in **Figures 6A–C**. Each plot in the top row shows an overlapping waveform (green) with its best-matched synthetic waveform (black). In the corresponding plot in the bottom row, the superposition waveform from which the synthetic waveform was extracted is plotted using a dotted black line, and the extracted synthetic waveform is plotted using a thick black line. The two single unit templates that were combined to generate the superposition waveform, and subsequently the synthetic waveform, are shown in the thin red and blue lines.

**Figure 6A** describes the first scenario. In this scenario, single units fired relatively far apart in time from each other, and the resulting overlapping waveform was considered an overlapping spike solely due to the minor differences in the waveform when compared to the single unit spikes. This can be deduced from the large phase shift between the single unit templates when assessing the superposition waveform from which the matching synthetic waveform was extracted (bottom plot in **Figure 6A**). It can be seen that for the synthetic waveform in this plot, the trigger point of the first template (blue waveform, Point 17) was outside the 32 points of the synthetic waveform (Point 32 to Point 63 of the superposition waveform), so the first template had only a small effect on the synthetic waveform. In this case, the overlapping waveform matched with this synthetic waveform should be assigned to the single unit cluster that mainly formed the synthetic waveform, which is the second template (red) in this example.

On the other hand, when two single units fired very close to each other in time, this resulted in overlapping waveforms deviating significantly from either of the single spikes (**Figures 6B,C**). As can be seen in the bottom row of **Figures 6B,C**, in contrast to Scenario 1, the trigger points of the two single unit templates that generated the superposition waveform were inside the 32 data points of the best-matching synthetic waveform, suggesting that the single units fired very close to each other in time to generate the overlapping waveform. In this case, it is possible that the single spikes occurred almost simultaneously, and as a result, the overlapping waveform only contained one minimum (**Figure 6B**, Scenario 2). It is also possible that even though the single unit spikes overlapped, the time shift was still large enough for two distinct minima, corresponding to the contributing spikes, to be identified in the overlapping waveform (**Figure 6C**, Scenario 3).

For Scenarios 2 and 3, the assignment was performed based on the number of local minima that exceeded the extraction threshold in the overlapping waveform. In order to do this, we used the same extraction threshold that was used initially to extract the spike waveforms from the high-pass filtered data. For example, in the top plot of **Figure 6B**, the arrow highlights the single local minimum in the overlapping waveform (shown in green), while in the top plot of **Figure 6C**, two local minima (highlighted by the arrows) exceeded the extraction threshold in the overlapping waveform. If more than one local minima in the overlapping waveform were identified (Scenario 3), the overlapping waveform was only assigned to one of the templates. This was because separate spikes were most probably extracted from the high-pass filtered data (corresponding to each local minimum) in the initial spike extraction phase, so we only needed to assign the current waveform to one single unit. The other waveform that was extracted, and potentially matched with some other synthetic waveform, will be assigned to its corresponding single unit cluster separately. In this scenario, the overlapping waveform was always assigned to the template with the trigger point that was closer to the trigger point of the synthetic waveform, as that single unit was probably the one that contributed the most to the overlapping waveform. In the example in **Figure 6C**, the overlapping waveform was assigned to the single unit corresponding to the blue template shown in the bottom plot, because the trigger point of the synthetic waveform (Point 42) was closer to the trigger point of the blue template (Point 43) rather than the trigger point of the red template (Point 35). In Scenario 2, only one local minimum was identified in the overlapping waveform, and this suggested that the two single units combined in such a way to create only one overlapping waveform, so we assigned the overlapping waveform to both single unit clusters.

### Final Processing

After adding the overlapping waveforms to their corresponding single unit clusters, we checked for refractory period violations. If the interval between two spikes was smaller than 1 ms, one of them was removed from the single-unit cluster and added to the multi-unit cluster. The waveform that was removed was the waveform with the smaller correlation coefficient when compared to the mean of the cluster. The final single-unit clusters after the overlapping waveforms had been assigned to the proper clusters are shown in **Figure 6D** for the sample electrode data, and **Figure 6E** for the sample tetrode data.

Other waveforms that were not clustered with any synthetic waveforms were grouped together and formed the multi-unit cluster (not shown in **Figures 6D,E**). The source code used in this method (written in MATLAB) is available for download from https://cortex.nus.edu.sg/sorting/.

### Evaluation

In order to assess the performance of our method, we tested our method on datasets with different signal to noise ratios using the simulated data from Quiroga et al. (2004), which was downloaded from their website^2^.

In this dataset, there were four sets of simulations, Easy1, Easy2, Difficult1, and Difficult2, each consisting of waveforms from three neurons, with Easy1 and Easy2 made up of clearly distinct waveforms, while Difficult1 and Difficult2 consisted of waveforms that were more similar in shape. In this paper, we only show the results for the Difficult1 and Difficult2 datasets. The Difficult1 and Difficult2 datasets were each composed of four different subsets with increasing levels of noise, ranging from standard deviations of 0.05 to 0.2 times the spike amplitude. The details on how these simulated data were generated can be found in Quiroga et al. (2004).

We sorted the data using our method, using the same wavelet coefficients that were used in the Quiroga study as our clustering features. We selected these coefficients manually, because we did not find the probability measure returned by the Lilliefors test, used in the Quiroga study, to be a reliable measure for choosing bi-modally distributed features when applied to our dataset. Using manual selection, we reduced the number of coefficients required to cluster the data to four coefficients with multimodal distributions instead of the ten non-normally distributed wavelet features in the Quiroga study.

In order to examine the performance of our method for additional datasets, we generated two simulated datasets using the waveforms from electrode and tetrode recordings, respectively that were previously performed in our lab. For the electrode data, extra-cellular recordings were performed using Tungsten microelectrodes in the primary visual cortex of behaving macaque monkeys (Friedman-Hill et al., 2000; Gray et al., 2007). For the tetrode recordings, the data were recorded in the striate cortex of anesthetized cats during the presentation of natural movies (Yen et al., 2007). In both datasets, the analog signals were digitized at a rate of 30 kHz on each channel.

We followed a method similar to that used by Quiroga et al. (2004) to generate the simulated data. For each recording, we first identified the template clusters, and averaged the waveforms in the template clusters to obtain the templates. Then, for each template, we generated a surrogate spike train using a method derived from Berry and Meister (1998). We made the following two changes to their method: (1) instead of deriving the recovery function from neuronal responses, we used a sigmoid function with a 5-s standard deviation; and (2) instead of deriving the free-firing rate from the PSTH obtained from the recordings, we generated random values from a uniform distribution in the interval of [0, 20] spikes per second. This method enabled us to generate spike trains that exhibited refractory periods that were more realistic compared to those exhibited by Poisson spike trains (Berry and Meister, 1998). Spike trains were generated for each template cluster independently (i.e., 2 template clusters for each of the 4 electrode recordings, and 3 template clusters for each of the 4 tetrode recordings), and each spike in the surrogate spike train was then replaced by the corresponding template. These spike trains were then added together to create the signal part of the synthetic raw data.

In order to generate noise in the synthetic data, we first generated spike trains that consisted of a mix of spike waveforms extracted from our data, and then scaled the signal amplitudes down to resemble noise. We did this by constructing 1000 other surrogate spike trains using a method similar to the above, except that we drew the free firing rate from a uniform distribution on the interval [0, 200], and instead of replacing spikes by templates, each spike was replaced by a waveform randomly picked from all the extracted waveforms in the recording. These waveforms consisted of single-unit, multi-unit, and overlapping waveforms. Next, we added these 1000 spike trains together, removed the mean value of the noise from the generated noise signal, and normalized the amplitude of the noise signal to 1 μV by dividing the noise signal by its maximum amplitude. We then scaled the noise by an amplification factor to equalize the standard deviation of the simulated noise with the standard deviation of the background signal in the actual recording. The simulated noise data was then added to the signal data generated above to form the final synthetic raw data.

For the electrode data, we selected four recordings, each with two template clusters, to generate four synthetic raw datasets. For the tetrode data, we selected four recordings with three single unit templates to generate four synthetic raw datasets. Each synthetic raw dataset was 30 min long with a sampling rate of 30 kHz. These simulated data for both electrode and tetrode are available at https://cortex.nus.edu.sg/sorting/Downloads.html.

For both our simulated dataset and that from the Quiroga study, we defined an overlapping waveform as a waveform that was generated by the overlap of two single units with a phase shift smaller than 1 ms.

In addition to simulated datasets, to assess the characteristics of our method when sorting real datasets, we sorted the real electrode and tetrode recordings described earlier, using our automated method.

## Results

The results of sorting the simulated data from the Quiroga study with Wave_clus (Quiroga et al., 2004), KlustaKwik, and our method are shown in **Table 1**. It can be seen that our method performed well in resolving single-unit and overlapping spike waveforms for this dataset at various signal-to-noise ratios. On average, our method was able to resolve an average of 83% of the overlapping waveforms, an improvement of approximately 32% over the results reported in the Quiroga study, and a 17% improvement over just using KlustaKwik.

**Table 1:** The results of applying Wave_clus (WC), KlustaKwik (KK), and our method (CM: current method) to simulated data from Quiroga et al. (2004).

Dataset	Spikes	Sorted (WC)	Sorted % (WC)	Sorted (KK)	Sorted % (KK)	Sorted (CM)	Sorted % (CM)
D1_0.05	3383 (365)	3193 (199)	94% (55%)	3017 (237)	89% (68%)	3349 (331)	99% (91%)
D1_0.1	3448 (317)	3313 (190)	96% (60%)	3127 (216)	91% (70%)	3409 (279)	99% (88%)
D1_0.15	3472 (343)	3183 (195)	92% (57%)	3114 (231)	90% (72%)	3393 (280)	98% (82%)
D1_0.2	3414 (313)	2767 (153)	81% (49%)	3013 (197)	88% (68%)	3238 (232)	95% (74%)
D2_0.05	3364 (349)	3137 (125)	93% (36%)	3014 (206)	90% (59%)	3337 (322)	99% (92%)
D2_0.1	3462 (271)	3326 (143)	96% (53%)	3146 (177)	91% (65%)	3416 (230)	99% (85%)
D2_0.15	3440 (372)	3055 (201)	89% (54%)	2848 (230)	83% (62%)	3151 (297)	92% (80%)
D2_0.2	3493 (351)	1983 (141)	57% (40%)	2759 (215)	79% (64%)	3022 (246)	87% (70%)
Average			87% (51%)		88% (66%)		96% (83%)

*The number in each column includes both single spikes and overlapping waveforms. The numbers for overlapping waveforms are shown in parentheses*.

In order to find out how well our method can be applied to different datasets, we also applied our method to simulated dataset that were generated using our electrode and tetrode recordings. The results of applying our method to these datasets are shown in **Tables 2**, **3**, respectively. Our method was able to resolve single-unit and overlapping waveforms with a high degree of accuracy for both the electrode (91%, with 0.19% false positives) and tetrode (95%, with 0.27% false positives) data. It should be noted that while the spike waveforms used to generate the simulated data varied significantly in shape and amplitude across datasets, we used one set of features to sort all the electrode datasets, and one set of features to sort all the tetrode datasets. These features were selected earlier to sort the real recordings based on subjective measures, and were not specifically selected to sort the simulated data. Also, we did not change any of the parameters for the clustering to sort different simulated datasets.

**Table 2:** The results of applying our method to the simulated electrode datasets.

Dataset	Spikes	Sorted	Sorted %	False positives
1	68394 (2435)	66099 (2136)	96% (87%)	49 (0.07%)
2	68662 (2463)	68386 (2357)	99% (95%)	5 (0.007%)
3	69086 (2489)	67330 (2238)	97% (89%)	320 (0.46%)
4	68341 (2651)	66911 (2496)	97% (94%)	159 (0.23%)
		Average	97% (91%)	0.19%

*The number listed in the second to the fourth column includes both single spikes and overlapping waveforms, with the numbers for overlapping waveforms shown in parentheses*.

**Table 3:** The results of applying our method to the simulated tetrode datasets.

Dataset	Spikes	Sorted	Sorted %	False positives
1	102382 (7396)	101776 (7157)	99% (96%)	122 (0.11%)
2	102743 (7467)	99987 (7234)	97% (96%)	170 (0.16%)
3	102106 (7344)	100452 (7088)	98% (96%)	130 (0.12%)
4	102488 (7499)	94640 (6870)	92% (91%)	719 (0.70%)
		Average	97% (95%)	0.27%

*The number listed in the second to the fourth column includes both single spikes and overlapping waveforms, with the numbers for overlapping waveforms shown in parentheses*.

**FIGURE 7:**
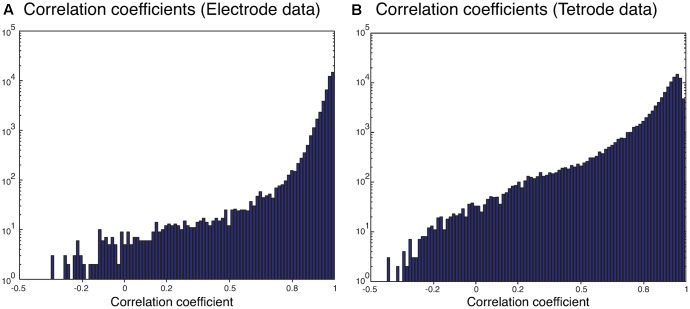
Correlation coefficients of the best matching synthetic waveforms to the overlapping waveforms. **(A)** Results from electrode data. **(B)** Results from tetrode data. Note the log-scale on the vertical axis.

We also sorted the real electrode and tetrode recordings mentioned above. The histograms of the correlation coefficients for the best matching waveform in the electrode and tetrode datasets are shown in **Figure 7**. We found a large proportion of the correlation coefficients were larger than 0.8 (96.66% for electrode data and 83.10% for tetrode data), suggesting that our algorithm was often able to match overlapping waveforms with the appropriate synthetic waveforms. However, in a small number of cases, the correlation coefficients were smaller than 0.2 (0.42% for the electrode data and 1.02% for the tetrode data) and even negative, showing that while in most cases the clustering performance was acceptable, occasionally waveforms were clustered inappropriately with other waveforms that looked significantly different.

**FIGURE 8:**
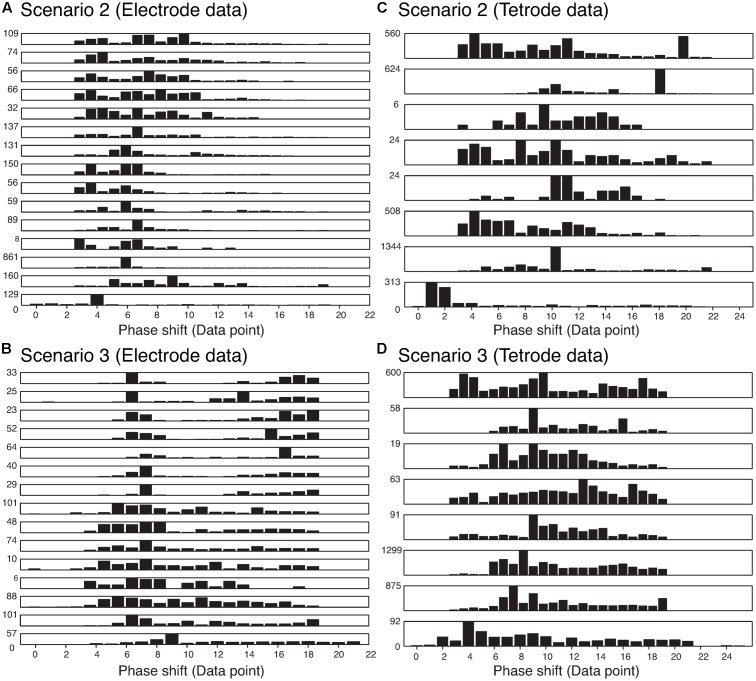
Distribution of the absolute value of the time differences in the trigger points in the best-matching synthetic waveforms in the two assignment scenarios in the electrode and tetrode data. Each row represents a single recording. **(A)** Distribution of trigger point differences when only one trigger point was found in the overlapping waveform (Scenario 2) in the electrode data. **(B)** Distribution of trigger point differences when two trigger points were found in the overlapping waveform (Scenario 3) in the electrode data. **(C)** and **(D)** Same as **(A)** and **(B)** but for tetrode data.

We also wanted to ensure that our method was not sensitive to the phase shift of the single spikes that constituted an overlapping waveform, and were able to resolve the spikes regardless of the amount of overlap. Thus, we measured the time difference between the trigger points of the two templates that made up each best-matching synthetic waveform. The histograms of the absolute value of these time differences are shown in **Figure 8**. We only included overlapping waveforms assigned in Scenarios 2 and 3 described above. Most of the cells exhibited a uniform distribution over a range of time differences (30 data points would be equivalent to 1 ms) indicating that the algorithm was able to recover overlapping waveforms over a range of time differences. On the other hand, some of the cells displayed remarkably precise time differences (e.g., the bottom-most and the 3rd-from-the-bottom histograms in Scenario 2 for electrode data). Considering the uniform distribution observed in other cells, we were confident that this effect was not due to a bias in the sorting process. For Scenario 2, because only one trigger point was found in the overlapping waveform, the trigger points were typically less than 12 points (i.e., 0.4 ms) apart. For Scenario 3, because two trigger points were found, the time differences typically spanned a greater range. Overall, we found 74.24% of the differences were smaller than 10 data points (i.e., 0.3 ms), while 13.84% of the differences were larger than 15 data points (i.e., 0.5 ms).

## Discussion

Our automated method for resolving overlapping waveforms produced good results when sorting the simulated datasets we studied. The main difference between our method and methods previously reported is that we used a clustering method to find the best match for each overlapping waveform among possible synthetic waveforms. This improved the performance, as we were able to use more than one feature to compare the overlapping waveforms with available templates. Also, unlike nearly all the methods we discussed in the Introduction, our method did not require setting a fixed threshold on similarity measures (e.g., correlation coefficients, root-mean-square error, etc.) to identify the synthetic waveforms that were similar to the overlapping waveforms, which makes it easy to apply this method to different datasets. Instead, we chose to let the clustering algorithm decide if waveforms belong together. While this implies that appropriate features have to be used during the clustering at this stage, we found in our experience that the same features used to identify clusters of single units can be used successfully to resolve overlapping waveforms.

Another advantage of our method was that we generated superpositions at all possible phase shifts, which gave us the ability to resolve overlapping waveforms that were generated by synchronous firing of two single units. Moreover, because we extracted the synthetic waveforms from these superpositions, instead of performing comparisons at all the data points, we were able to substantially increase the efficiency of our method. Unlike a number of other methods used in overlap resolution (Lewicki, 1998; Zhang et al., 2004), we have also made no assumptions about the noise or the distribution of neuronal activity.

In addition, our method makes use of software packages like KlustaKwik and MClust, which are already in widespread use in many electrophysiology laboratories around the world. This makes it easier for our method to be integrated into the spike sorting workflow in most situations than if, for instance, SVM, neural networks, Hidden Markov Models, or linear filters were to be added.

While resolving overlaps was automated and unsupervised, the template cluster selection using measures of cluster isolation and similarity was performed manually in our approach. However, as the resolution of overlapping waveforms is independent of how the templates are selected, other potential automated methods could be used to choose the single-unit templates.

Although one of the advantages of our method is that we avoid using a fixed threshold to assign the overlaps to suitable single units, or exclude noise from our assignments, it does not imply that no parameters need to be set. Suitable features and clustering method still have to be selected carefully, and may vary from dataset to dataset. In addition, the clustering algorithms have different parameters that must be tuned properly. However, since these typically need to be done to identify single units in the first place, our method simply reuses these same parameters in resolving overlapping waveforms, and does not introduce additional parameters that have to be fine-tuned.

Our method, however, cannot deal effectively with differences in spike waveforms from a single cell caused by electrode movement or bursting cells (Gray et al., 1995). Currently, these have to be identified and dealt with manually, although any clustering method that addresses these problems can be used instead of the KlustaKwik software package that is used here, which do not address this issue. Also, an incremental method as suggested by Ekanadham et al. (2014) to address the same issue with their method, or a new method proposed by Shan et al. (2017), may be good candidates to sort spikes that exhibit shape and amplitude changes as a result of tissue relaxation or movement of the electrode as time passes.

Also, we should mention another method that has several advantages over our method. This method, described in Herbst et al. (2008), uses a template matching approach that finds the combination of templates with the smallest mean square error relative to each spike using Hidden Markov Models. Herbst et al. (2008) obtained better results compared to our method in sorting the Quiroga et al. (2004) dataset. The main advantage of this method over our method, and all the other methods described earlier, is that the method can be used to automatically detect the templates. However, the threshold (*p*-value related to firing probability of the cells) learned in the learning phase usually has to be adjusted in order to sort the spikes, because otherwise the rate of false positive will be relatively high. They proposed a mathematical equation to guess the proper *p*-value, but when we applied the proposed equation to our experimental dataset, we still needed to lower the threshold based on the observed results until we obtained acceptable results. Along with some of the newer sorting methods for high density multi-electrode arrays that are available to resolve overlapping waveforms (Marre et al., 2012; Pachitariu et al., 2016; Rossant et al., 2016; Yger et al., 2017), it will be interesting to perform a comparison between these various methods for resolving overlapping waveforms in a future study.

## Author Contributions

All authors contributed significantly to developing and implementing the method and revising the paper. YM and S-CY analyzed the data and drafted the manuscript.

## Conflict of Interest Statement

The authors declare that the research was conducted in the absence of any commercial or financial relationships that could be construed as a potential conflict of interest.

## Footnotes

1 http://redishlab.neuroscience.umn.edu/MClust/MClust.html

2 http://www2.le.ac.uk/departments/engineering/research/bioengineering/neuroengineering-lab/spike-sorting
